# Renal Cell Carcinoma Presenting as Syncope due to Saddle Pulmonary Tumor Embolism

**DOI:** 10.3390/diseases10040119

**Published:** 2022-12-02

**Authors:** Mohamad K. Elajami, Ephraim Mansour, Hisham F. Bahmad, Gerard Chaaya, Steven DeBeer, Robert Poppiti, Yumna Omarzai

**Affiliations:** 1 Department of Internal Medicine, Mount Sinai Medical Center, Miami Beach, FL 33140, USA; 2 Arkadi M. Rywlin M.D. Department of Pathology and Laboratory Medicine, Mount Sinai Medical Center, Miami Beach, FL 33140, USA; 3 Division of Hematology and Medical Oncology, Department of Internal Medicine, Mount Sinai Medical Center, Miami Beach, FL 33140, USA; 4 Division of Cardiac Surgery, Mount Sinai Heart Institute, Columbia University, Miami Beach, FL 33140, USA; 5 Department of Translational Medicine, Florida International University, Herbert Wertheim College of Medicine, Miami, FL 33199, USA

**Keywords:** case report, renal cell carcinoma, saddle pulmonary embolism, tumor embolism

## Abstract

Pulmonary embolism (PE) is defined as the obstruction of the pulmonary artery or one of its branches by a blood clot, tumor, air, or fat emboli originating elsewhere in the body. A saddle PE occurs when the obstruction affects the bifurcation of the main pulmonary artery trunk. We present a case of a 46-year-old man who presented to our hospital due to an episode of syncope. Computed tomography angiography (CTA) of the chest showed extensive PE and abdominal CT scan showed a large 8 cm left renal mass with inferior vena cava (IVC) thrombus. Emergent embolectomy, left total nephrectomy, and IVC tumor removal were performed yielding the diagnosis of clear cell renal cell carcinoma (RCC). Interestingly, our patient did not experience any symptoms related to his RCC until the diagnosis of PE due to syncope, and the asymptomatic tumor was found out to be the possible cause of this PE due to the presence of tumor cells constituting the tumor embolus. It is thus recommended to improve the early screening process for RCC. Besides, clinicians should pay attention to patients presenting with uncharacteristic symptoms of RCC who might present with symptoms of saddle PE.

## 1. Introduction

Comprising one of the most common presentations in the emergency department, pulmonary embolism (PE) is counted as the third most fatal cardiovascular pathology worldwide [1,2]. Etiologically, it is caused by the blockage of the pulmonary artery and/or its segmental and subsegmental branches, by blood clots arising mainly in the venous system of the lower extremities induced by Virchow triad: stasis of blood flow, vascular endothelial injury, and hypercoagulability [3]. PE can be also caused by air, tumor, or fat emboli [4]. Clinically, patients present with dyspnea, hypoxia, tachypnea, and/or tachycardia. In severe untreated cases or in case of massive PE, the patient may present with hemodynamic instability and sudden death [2].

Cancer can manifest as a coagulation disorder with its associated sequela [5]. Some malignancies are more often associated with thrombotic complications such as pancreatic, stomach, bladder, renal, and lung cancers [6,7]. Blood coagulation is triggered by tumor cells that commonly express tissue factor (TF), a procoagulant molecule and cellular initiator of the coagulation cascade [8,9]. Tumors including renal cell carcinoma (RCC) can invade the inferior vena cava (IVC) and develop PE [10]. This condition causes very unstable hemodynamics and is often fatal.

We present a case of a 46-year-old man with a history of chronic anemia who presented with a syncopal episode. Workup revealed a saddle PE most likely originating from a left-sided RCC that was invading the left renal vein and inferior vena cava. Emergent IVC embolectomy and left total nephrectomy were performed. Pathologic examination yielded the diagnosis of clear cell RCC with tumor thromboembolus. This surgical case report was conducted and reported in accordance with Surgical CAse REports (SCARE) guidelines for reporting case reports.

## 2. Case Presentation

A 46-year-old man with a medical history of bronchial asthma and chronic anemia presented to the emergency department of our institution complaining of shortness of breath and an episode of syncope. The patient stated that he had experienced presyncopal episode while he was in the bathroom. No head trauma was reported by the patient. Shortness of breath started a few hours prior to the syncopal episode and were exacerbated with physical exertion and relieved with rest. The patient also reported a history of night sweats, decreased appetite, and unintended 20 pounds weight loss during a 3-month duration. Surgical history was significant for left total hip arthroplasty post fracture (in 2019) and testicular torsion surgery (in infancy). No relevant family history was present. The patient reported, however, being allergic to ketoprofen.

Upon presentation, the patient’s vital signs were normal: arterial blood pressure was 104/83 mmHg, pulse was 101 beats per minute, respiratory rate 20 per minute, temperature 36.7 °C, and SpO_2_ 100% on 6-Liter nasal cannula. Body mass index (BMI) was 21.41 kg/m². Physical examination was noncontributory. Laboratory results are summarized in Table 1. Electrolyte levels were within normal limits. High sensitivity troponin was elevated at 364 pg/mL (reference range 4–79 pg/mL) and peaked at 2935 mg/mL few hours after presentation, and then trended down, suggestive of non-ST elevation myocardial infarction (NSTEMI). Urinalysis was notable for trace hematuria and proteinuria

**Figure 1:**
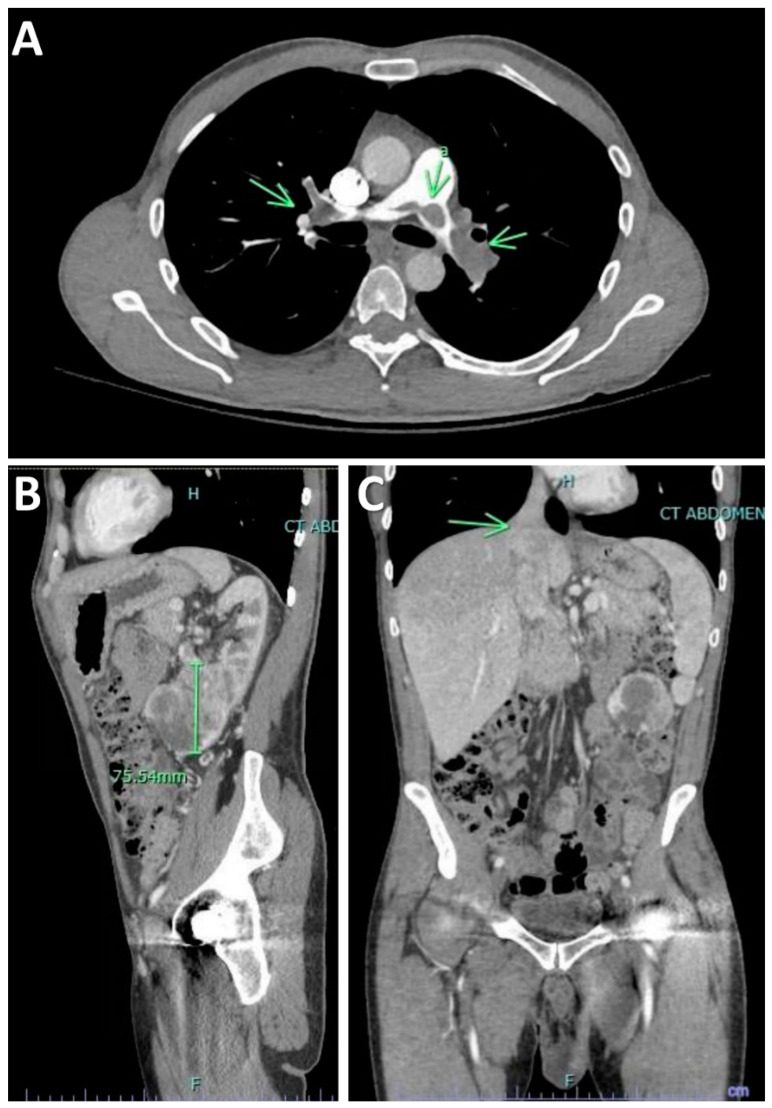
Imaging studies. (**A**) Computerized tomography angiogram (CTA) of the chest was notable for extensive filling defects consistent with saddle pulmonary embolism. (**B**) Abdominal CT scan revealed 6.5 × 8.0 × 7.6 cm heterogenous mass at the left inferior pole abutting Gerota’s Fascia, concerning for renal cell carcinoma, clear cell type. (**C**) Tumor thrombus was also seen in the left renal vein and inferior vena cava, extending to the level of the diaphragm.

A computerized tomography angiogram (CTA) of the chest was notable for extensive filling defects extending along the bifurcation of the main pulmonary artery consistent with saddle pulmonary embolism, extending into the main, lobar segmental and subsegmental pulmonary arteries, with essentially occlusive emboli noted throughout the entire left lower lobe pulmonary arterial system (Figure 1A). There was also evidence of elevated right heart pressure with asymmetric right atrial (RA) and right ventricular (RV) enlargement with mild flattening of the interventricular septum and RV: left ventricular (LV) ratio of 1.54. Echocardiography demonstrated a LV ejection fraction (LVEF) between 60–65% with hypokinesis of the right ventricular free wall and hypercontractility of the RV apex (McConnell’s sign). A pedunculated-like echogenicity in the inferior vena cava (IVC) was observed, with the longest segment measuring 8.5 cm and the distal round portion measuring 2.3 × 2.6 cm terminating around 7 cm proximal to the IVC/RA junction. The RV systolic pressure was estimated to be at least 38 mmHg with a D-shaped interventricular septum during systole and diastole consistent with RV pressure and volume overload. Those findings were suggestive of right heart strains, which are encountered in the clinical milieu of massive PE. Duplex venous ultrasound of the lower extremities showed a nonocclusive deep vein thrombosis (DVT) in the proximal and mid segments of the right femoral vein. The patient was urgently started on heparin anticoagulant 25,000 unit/250 mL in 0.9 % sodium chloride IV solution.

The patient was also being followed up in the hematology clinic at our institution after he was referred by his primary care physician for a chronic history of fatigue secondary to anemia. As a part of the anemia workup, iron studies were remarkable for low iron levels of 17 mcg/dL (reference range 50–180 mcg/dL), total iron binding capacity (TIBC) of 226 mcg/dL (reference range 250–425 mcg/dL), low iron saturation of 8% (reference range 20–48%) and high ferritin level of 487 ng/mL (reference range 38–380 ng/mL), consistent with anemia of chronic disease. He had an elevated ESR at 103 mm/hr (reference range ≤ 15 mm/h) and CRP at 172.3 mg/L (reference range <8.0 mg/L).

Abdominal CT scan revealed a 6.5 × 8.0 × 7.6 cm heterogenous mass at the left inferior pole abutting Gerota’s fascia, with central areas of necrosis and peripheral nodular avid enhancing components, without intraperitoneal extension, concerning for RCC, clear cell type (Figure 1B). Tumor thrombus was seen in the left renal vein and inferior vena cava, extending to the level of the diaphragm (Figure 1C).

**Figure 2:**
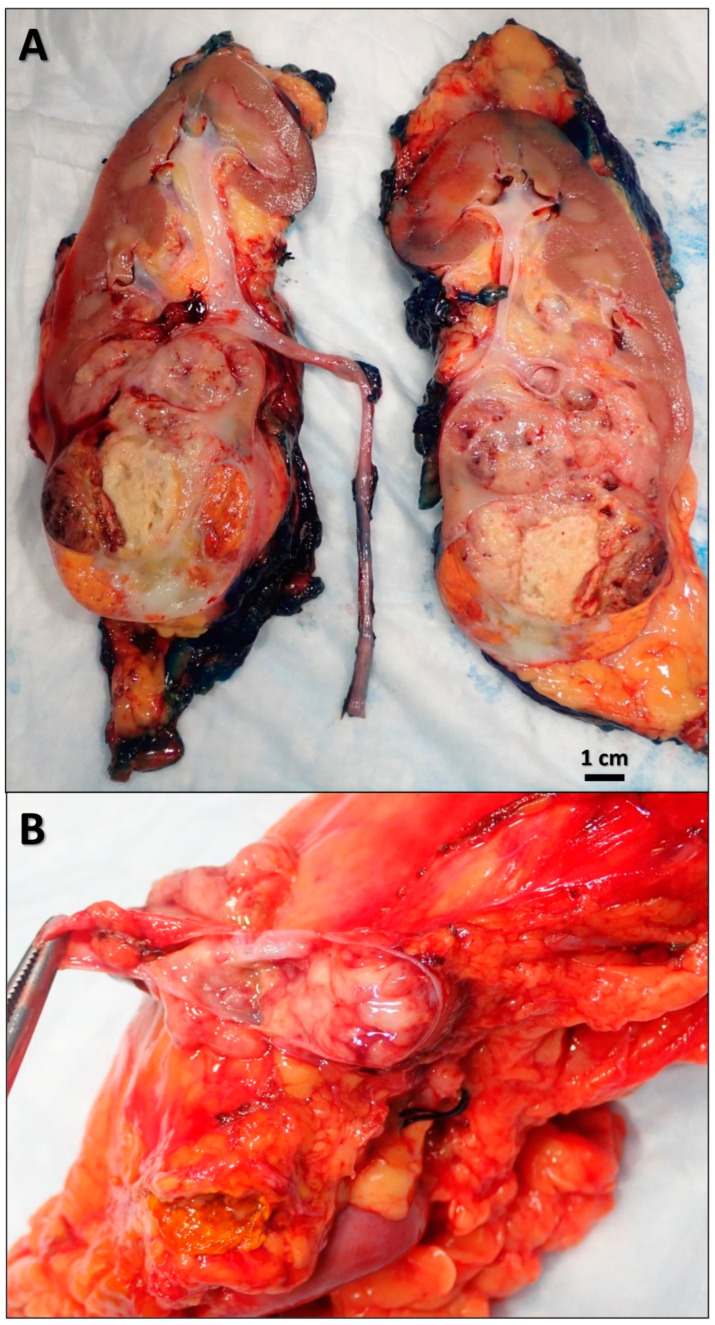
Gross image of the left kidney mass. Gross examination revealed a left kidney with a lobulated mass bulging from the inferior pole measuring 7.6 × 6.2 × 5 cm with a bright yellow, focally hemorrhagic cut surface and areas of central scarring (**A**), grossly invading the renal vein (**B**).

**Figure 3:**
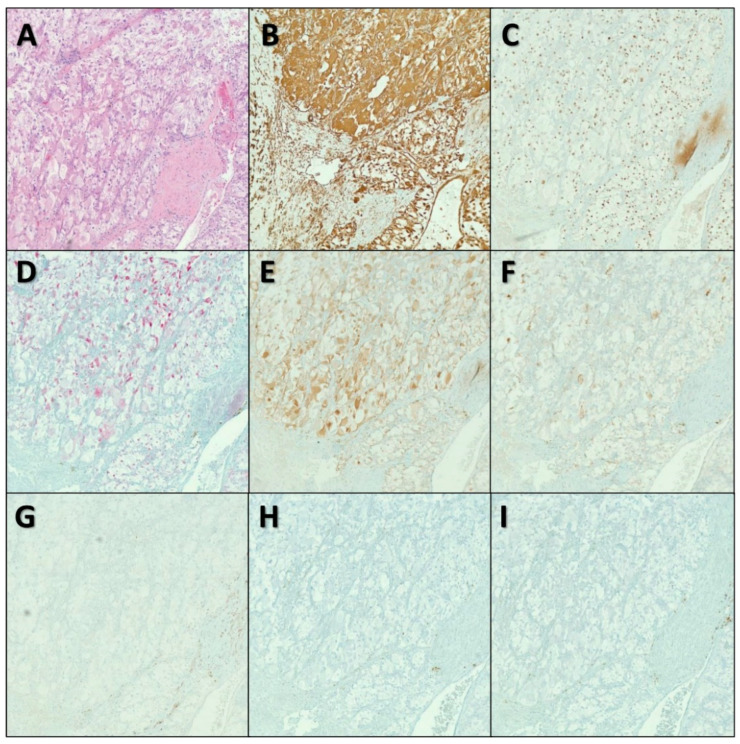
Microscopic images of the renal mass. Microscopic examination showed that the tumor is composed of solid sheet of neoplastic cells with clear cytoplasm and delicate branching vasculature. A diagnosis of clear cell renal cell carcinoma with rhabdoid features was made (**A**). Immunohistochemical stains were positive for Vimentin (**B**), PAX-8 (**C**), Racemase (p504s) (**D**), AE1/AE3 (focally) (**E**), EMA (weak, focally) (**F**), while negative for CK7 (**G**), HMB-45 (**H**), and Melan-A (**I**). Microscopic images were examined at 200× objective.

**Figure 4:**
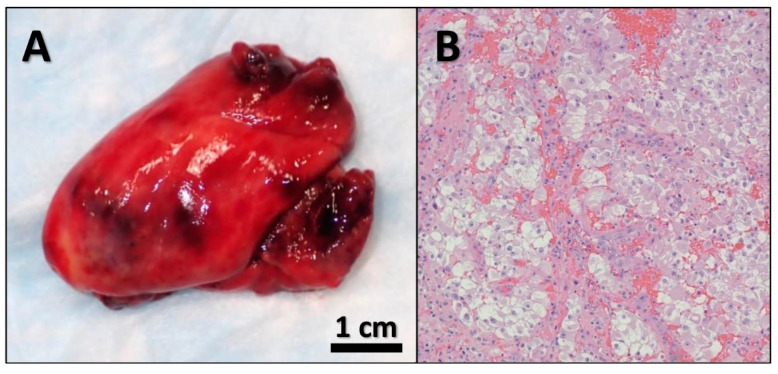
Gross and microscopic images of the left inferior vena cava tumor thrombus. (**A**) Gross image of the tumor thrombus. (**B**) Microscopic examination of the tumor thrombus showed renal cell carcinoma cells with clear cell and rhabdoid features admixed with clotted blood. Microscopic images were examined at 200× objective.

For the aforementioned findings, a multidisciplinary team including a urologist, a cardiothoracic surgeon, and a vascular surgeon were consulted for emergent surgical intervention. The patient was taken to the operating room, and he underwent emergent thoracotomy with pulmonary embolectomy and left radical nephrectomy with renal vein thrombectomy. Pathology revealed a left kidney with a lobulated mass bulging from the inferior pole measuring 7.6 × 6.2 × 5 cm with a bright yellow, focally hemorrhagic cut surface and areas of central scarring (Figure 2). Microscopic examination showed that the tumor was composed of solid sheets of neoplastic cells with clear cytoplasm and delicate branching vasculature. A diagnosis of clear cell RCC with rhabdoid features (approximately 30%) and extensive necrosis (approximately 40–50%) was made. The tumor histologic grade (according to the World Health Organization (WHO)/International Society of Urological Pathology (ISUP) grading system) was G4 (extreme nuclear pleomorphism and/or multi-nuclear giant cells and/or rhabdoid and/or sarcomatoid differentiation). Pathologic stage (according to the American Joint Committee on Cancer (AJCC) 8th Edition) was pT3cN0M1 (tumor extends into vena cava above the diaphragm or invades the wall of the vena cava; no regional lymph node metastases; distant metastases present in pulmonary artery and inferior vena cava). Special immunohistochemical (IHC) stains supported our diagnosis (Figure 3). The tumor was found to be invading the renal sinus and renal vein. The left IVC tumor thrombus also showed RCC cells with clear cell and rhabdoid features (Figure 4). Fluorescence in situ hybridization (FISH) analysis was performed on the tumor tissue for *TFE3* rearrangement yielding negative results. The patient was extubated 2 days after the surgery. He was transferred to the floor and subsequently discharged to inpatient rehabilitative services. On discharge, the patient was prescribed Apixaban 5 mg (one tablet by mouth every 12 h) and Aspirin Enteric Coated 81 mg (one tablet by mouth daily).

Genetic analysis testing of the patient’s blood was performed through INVITAE multi-cancer panel (Invitae Corp., San Francisco, CA, USA). The Invitae Multi-Cancer Panel analyzes 84 genes associated with genetic disorders and hereditary cancers across major organ systems. Results failed to reveal any pathogenic variants known to cause clear cell RCC. Besides, Guardant360^®^ CDx testing (Guardant Health, Redwood City, CA, USA) was performed on the patient’s blood to provide tumor mutation profiling for his advanced malignancy. Guardant360^®^ CDx sequences 74 cancer-associated genes to identify somatic alterations. Cell-free DNA (cDNA) is extracted from plasma, enriched for targeted regions, and sequenced using the Illumina platform and hg19 as the reference genome. No circulating tumor cDNAs were identified. Lastly, Caris complete molecular profiling was performed on the patient’s tumor tissue (CARIS Life Sciences, Phoenix, AZ 85040, USA) revealing a pathogenic *VHL* mutation (Exon 1–p.S68). Other cancer-type relevant biomarkers identified are summarized in Table 2.

At follow-up after 9 months, the patient was doing well at home, on a combination therapy of a tyrosine kinase inhibitor (Cabozantinib) and immunotherapy (Nivolumab) for his advanced clear cell RCC disease, besides chronic dual anticoagulation therapy of Apixaban and Aspirin. This treatment regimen is as a result of a randomized, open-label clinical trial on 651 patients (CheckMate -9ER Trial in First-Line Advanced Renal Cell Carcinoma; Funded by Bristol Myers Squibb and others; ClinicalTrials.gov number, NCT03141177). Results from this trial showed that Nivolumab plus Cabozantinib had significant benefits over Sunitinib with respect to progression-free survival, overall survival, and likelihood of response in patients with previously untreated advanced RCC [11].

## 3. Discussion

Historically, the coexistence of cancer and thrombosis was initially studied in 1865 by Armond Trousseau, a French clinician, who described several cases of migratory thrombophlebitis and its association with visceral malignancies [12]. Venous thromboembolism incidence varies among cancer types, with a higher incidence occurring in metastatic stage disease, and this has been associated with poor prognosis among patients [13].

Among the most commonly diagnosed urogenital malignancies, renal cell carcinoma (RCC) is the third on the list, with 400,000 newly diagnosed cases and nearly 180,000 deaths reported in 2020 [14]. Clear cell RCC accounts for the majority of cases (75–80%), followed by papillary (10–15%), and chromophobe (5%) RCC [15]. The classic symptomatology triad of patients with suspected RCC includes flank pain, hematuria, and a palpable abdominal mass. Some patients present with nonspecific symptoms such as weight loss, fatigue, or anemia [16]. On rare occasions, RCC invades the ipsilateral renal vein (33%) or inferior vena cava (6%) with subsequent development of cardiopulmonary complications such as hemodynamic instability and pulmonary embolus [17]. In a study by Li et al., authors described a case of a 43-year-old man who presented with ‘squeezing’ chest pain mimicking acute coronary syndrome and was subsequently diagnosed with pulmonary embolism, which led to the detection of clear cell RCC [18]. Similarly, in our case, the patient presented with syncope due to a saddle pulmonary embolism and was consequently diagnosed with clear cell RCC.

Due to the anatomical proximity of renal tumors to the renal sinus, RCC can locally invade the venous structures within the sinus with a tumor thrombus that can propagate distally into the inferior vena cava (IVC), then the right atrium and pulmonary arteries [19]. This might be applicable to our case. However, pulmonary tumor embolism can occur when tumors seed the circulation with individual cells or clusters of cells that travel reaching the pulmonary vasculature. This can either cause obstruction of the microvasculature or activation of the coagulation system and concomitant thrombotic obstruction [20]. It is possible that the saddle embolus originated in the lower extremity veins, and not from the kidney tumor thrombus in our case. In very rare cases, kidney tumor thrombus detaches and presents as sudden dyspnea or sudden death due to a large embolus obliterating the pulmonary arteries. In our patient, it is probable that the saddle embolus was caused by detachment of a thrombus from the lower extremities and further activation of the coagulation system by circulating tumor cells occurred.

Venous invasion is a poor prognostic factor in RCCs, leading to distant metastases [21,22]. According to the American Joint Committee on Cancer (AJCC) staging manual of renal malignancies, venous invasion is designated as pathologic stage T3, whether the invasion is into the segmental or main renal vein [23]. Histologically, segmental renal veins are present in the renal sinus, and hence, any invasion of the renal sinus fat, lymphatics or venous structures by tumor cells is thought to contribute to hematogenous and lymphatic spread from RCC [24].

There are different management modalities that can be employed in treating pulmonary embolism with RCC. The first case was described in 1977 by Daughtry et al., where the patient underwent surgical embolectomy, as in our case [25]. Thrombolysis is also an option. However, the definitive treatment of RCC in the presence of intravascular tumor is surgical resection of the tumor and thrombectomy to improve the long-term survival of patients and prevent recurrent emboli and further extension of the tumor embolus [26].

## 4. Conclusions

In conclusion, pulmonary tumor emboli are rare yet should be taken into consideration and be included in the differential diagnosis of patients with a history of malignancy who present with dyspnea, hypoxia, cough, or chest pain. It is also recommended to improve the early screening process for RCC, and clinicians should pay attention to patients presenting with uncharacteristic symptoms of RCC who might present with symptoms of saddle PE, as in our case.

## Figures and Tables

**Table 1:** Relevant laboratory results of the patient.

Blood Test	Patient Value	Reference Range
White blood cell (WBC) count	23.9 × 10^3^/μL	4.8–10.8 × 10^3^/μL
Segmented neutrophils	88.6%	42–75%
Absolute neutrophil count	21.20 × 10^3^/μL	1.8–7.2 × 10^3^/μL
Red blood cell (RBC) count	3.71 × 10^6^/μL	3.93–5.22 × 10^6^/μL
Hemoglobin	9.2 g/dL	12.0–16.0 g/dL
Hematocrit	31.7%	37.0–47.0%
MCV	85.4 fL	79.0–92.2 fL
MCH	24.8 pg	25.6–32.2 pg
MCHC	29 g/dL	32.0–36.0 g/dL
Platelet count	387 × 10^3^/uL	150–450 × 10^3^/uL
Serum creatinine level	1.16 mg/dL	0.55–1.02 mg/dL
Blood urea nitrogen (BUN) level	12.0 mg/dL	7–18 mg/dL
Alkaline phosphatase level	401 U/L	46–116 U/L
Lactate level	6.6 mmol/L	0.4–2.0 mmol/L
Fibrinogen	781 mg/dL	200–400 mg/dL
Prothrombin time (PT)	17.9 s	12.4–15.2 s
INR	1.5	0.1–1.1
AP thromboplastin time (PTT)	56.5 s	24.7–39.8 s
pH	7.48	7.35–7.45
PCO2	29 mmHg	35.0–45.0 mmHg
PO2	190 mmHg	75.0–100.0 mmHg
HCO3	21.6 mmol/L	22.0–26.0 mmol/L

**Table 2:** Results of the genetic analysis testing of the patient’s tumor tissue performed through CARIS complete molecular profiling.

Detected Alteration(s)/Biomarker(s)	Method	Analyte	Result
*PBRM1*	Seq.	DNA-Tumor	Pathogenic Variant Exon 9–p.1287fs
PD-L1 (SP142)	IHC	Protein	Positive (90%)
*TSC1*	Seq.	DNA-Tumor	Pathogenic Variant Exon 9–p.1262fs
*MSI*	Seq.	DNA-Tumor	Stable
Mismatch Repair Status	IHC	Protein	Proficient
*TMB*	Seq.	DNA-Tumor	Low
*BAP1*	Seq.	DNA-Tumor	Variant of uncertain significance Exon 9–p.N229H

Abbreviations: IHC: immunohistochemistry; MSI: microsatellite instability; Seq.: sequencing; TMB: Tumor mutational burden.

## Data Availability

Not applicable.
